# A Critical Overview of ASP and Future Perspectives of NASP in EOR of Hydrocarbon Reservoirs: Potential Application, Prospects, Challenges and Governing Mechanisms

**DOI:** 10.3390/nano12224007

**Published:** 2022-11-14

**Authors:** Rasan Sarbast, Namam Salih, Alain Préat

**Affiliations:** 1 Department of Petroleum Engineering, Faculty of Engineering, Soran University, Soran 44008, Kurdistan Region, Iraq; 2 Research Group, Biogeochemistry & Modelling of the Earth System, Université Libre de Bruxelles, 1050 Brussels, Belgium

**Keywords:** nano-alkaline-surfactant-polymer, ASP, EOR, interfacial tension, contact angle, core-flooding

## Abstract

Oil production from depleted reservoirs in EOR (Enhanced Oil Recovery) techniques has significantly increased due to its huge demands in industrial energy sectors. Chemical EOR is one of the best approaches to extract the trapped oil. However, there are gaps to be addressed and studied well for quality and cost consideration in EOR techniques. Therefore, this paper addresses for the first time a systematic overview from alkaline surfactant polymer ((ASP)) and future perspectives of nano-alkaline surfactant polymer ((NASP)), its synergy effects on oil recovery improvement, and the main screening criteria for these chemicals. The previous findings have demonstrated that the optimum salinity, choosing the best concentration, using effective nano-surfactant, polymer and alkaline type, is guaranteed an ultra-low IFT (Interfacial Tension). Core flood results proved that the maximum oil is recovered by conjugating nanoparticles with conventional chemical EOR methods (surfactant, alkaline and polymer). This work adds a new insight and suggests new recommendation into the EOR application since, for the first time, it explores the role and effect of nanotechnology in a hybrid with ASP. The study illustrates detailed experimental design of using NASP and presents an optimum micro-model setup for future design of NASP flow distribution in the porous media. The presence of nano along with other chemicals increases the capillary number as well as the stability of chemicals in the solution and strengthens the effective mechanisms on the EOR.

## 1. Introduction

The United States energy information administration has forecasted that worldwide energy use will surge 28% by 2040. Hydrocarbons are regarded as the vital source of energy in the world [1]. Hydrocarbons are recovered in three stages: primary, secondary, and tertiary. Primary oil recovery or natural drive means production of oil due to a change in the production well pressure [2]. Secondary oil recovery starts when the pressure inside the well drops decreases significantly. In the secondary oil recovery reservoir, pressure increased due to fluid (water or gas) injection [3]. Tertiary recovery is used after the secondary stage to displace the trapped oil. Around 33% STOOIP (Stock Tank Oil Original in Place) recovered after primary and secondary methods [4]. After the secondary stage, high oil saturation remains in the reservoir formation [5]. Many oil companies focused on developing new technology at the beginning of 1980; in the US, oil production was expedited after using chemical flooding [6].

Chemical injection as the main EOR process mobilizes the remaining residual oil saturation by improving oil microscopic/macroscopic displacement efficiency [7,8]. Oil microscopic displacement efficiency is improved by surfactant and alkaline injection. Alkaline enhances the trapped oil mobility by adjusting the pH of in situ water [2]. On the other hand, oil macroscopic displacement efficiency is improved by polymer injection. All of the mentioned CEOR (Chemical Enhance Oil Recovery) methods aim to expedite the ultimate oil recovery.

The main limitation of CEOR method is the excessive adsorption at the rock surface. When these chemicals are introduced into the formation for oil recovery optimization, an extreme amount of surfactant and polymer is lost on the rock surface. This challenge makes the process very costly and have low efficiency in wettability alteration and IFT reduction.

Recently, nanoparticles were used as an effective chemical EOR method to reduce surfactant and polymer adsorption, to increase oil recovery by modifying wettability, and to reduce oil viscosity and water oil IFT. Recent works have suggested that using ASP in hybrid effectively improves residual oil production in the reservoir pore throats [9]. Recent advances, methods and technologies of these chemicals have not been summed up in detail. Therefore, a critical review on the role and effect of different types and concentrations of surfactant, polymer, alkaline and hybrid on oil recovery is needed

The main objective of our work is focused on the application of nano-alkaline surfactant polymer in EOR by considering the governing mechanisms of recovery and reservoir conditions. Moreover, the effect and role of hybrid nano-ASP flooding in carbonate and sandstone reservoirs are studied effectively by addressing different EOR mechanisms such as interfacial tension, wettability alteration and mobility control. Accordingly, the following questions would be addressed: (i) Could the new idea and recommendation by utilizing NASP design be successful in providing the multi-functional EOR agents? (ii) What are the advantages and disadvantages of using natural based surfactants in NASP over chemical surfactants, and which kind of surfactant is environmentally-friendly; (iii) What are the main challenges and limitations of ASP? (iv) What are the effects of different parameters on interaction between rock/fluid and fluid/fluid such as concentration and type of EOR agents, operational conditions such as salinity and temperature, and pressure of reservoir and injection conditions on the CEOR efficiencies? (v) How NASP can boost the oil recovery in real field projects? (vii) What are the main governing mechanisms of NASP in microscopic and macroscopic scales? Lastly, the authors will investigate the best scenario or method for guaranteeing the maximum oil recovery through NASP injection.

## 2. The Mechanisms and Role of NASP in CEOR

The EOR project is hugely controlled by mobility of fluids in the pore spaces. Favorable mobility (M) is achieved through reducing the mobility ratio to less than 1.0. Unstable displacement front occurs if M > 1.0. In this case, the large contrast in oil and water viscosity (displacing phase) fingers into oil (displaced phase) leads to premature breakthroughs at production wells, decreasing oil production. To overcome the viscous fingering issue, a polymer is used to enhance the viscosity of the displacing phase to decrease the mobility of the displaced phase (Equation (1)):

**M=(K_rw_/K_ro_) × (μ_o_/μ_w_)** (1)

where K_rw_ = water relative permeability, K_ro_ = oil relative permeability, µ_w_ = water viscosity, µ_o_ = oil viscosity. For microscopic sweeping, efficiency using AS (Alkaline Surfactant) has been utilized, in situ soap formation by alkaline flooding is not high enough to reduce IFT (Interfacial Tension) significantly [10]. The concept of using surfactant applies to washing surfaces of the oil reservoir rocks when these oils were trapped due to capillary pressure. ASP synergism is one of the successful CEORs in developing oil recovery in complex reservoir conditions. Moreover, it is cost effective in reservoirs that rely on gravity and imbibition in recovering the oil. Around 32 ASP field projects are surveyed in the literature [11]. Twenty-one field projects were reported in China followed by USA, India, Canada and Venezuela with 7, 2, 1 and 1 projects, respectively. Only one project was performed in an offshore reservoir, which was in Lagomar, Venezuela. Five-spot patterns were used in most of the ASP field projects [11]. Heavy alkylbenzene sulfonate was used in most of the projects. In these projects, pump working life and lifting system were damaged due to scale and corrosion. Due to scaling issues, weak alkali is widely used instead of strong alkali. The utilized surfactants were ORS-41HF, ORS-62, biosurfactant local Chinese surfactant product (OP10, KPS-1 and CY1), anionic surfactant (BES), (local petroleum sulfonate (YPS-3A), isobutanol, n-butanol, and isopropyl alcohol (cosurfactant). The main polymer used in an ASP slug was HPAM, 1275A, 3530S, and Pusher 700 [11]. Biopolymer (xanthan) was used only in one project [11].

**Figure 1:**
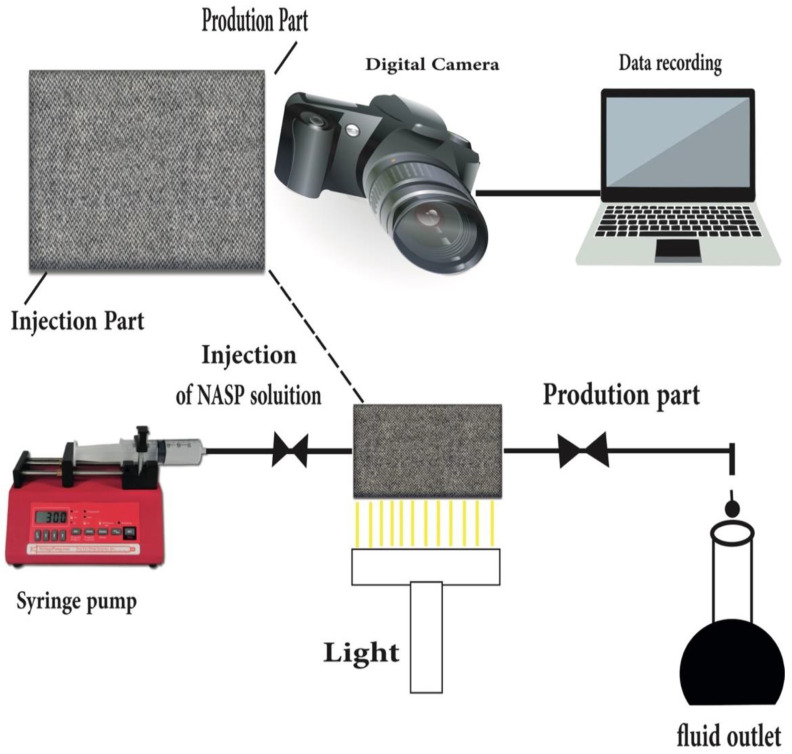
Future micromodel set up using NASP.

Table 1 depicts a summary of previous ASP works while highlighting the role and mechanisms of each chemical alone and in hybrid for enhancing oil mobilization. To enhance the mobility and investigate the role of the injected NASP, the volumetric sweeping efficiency micromodel as a future new insight in EOR would have been a new recommendation for oil companies. A micromodel experiment (Figure 1) is a future planned model to investigate the mechanism of the fluid flow on porous media via flow visualization, fluid interactions, pore space geometry and heterogeneity effects. To carry out the test, the oil–wet glass micromodel would be saturated with oil, followed by the injection of the prepared hybrid nano-ASP solution. The micromodel is placed horizontally to avoid the gravity effect. During the tests, high resolution pictures will reveal the fluid distribution in the micromodel taken at various time intervals.

From the previous lab and experimental works, it is quite clear that, for mobilizing and improving the trapped oil effectively and efficiently, most of the EOR mechanisms such as mobility, IFT, and wettability should be controlled and activated. For this objective, the ASP synergism effect should be addressed extensively and optimum concentration of each chemical should be selected for boosting the oil recovery.

## 3. Natural Surfactants

**Figure 2:**
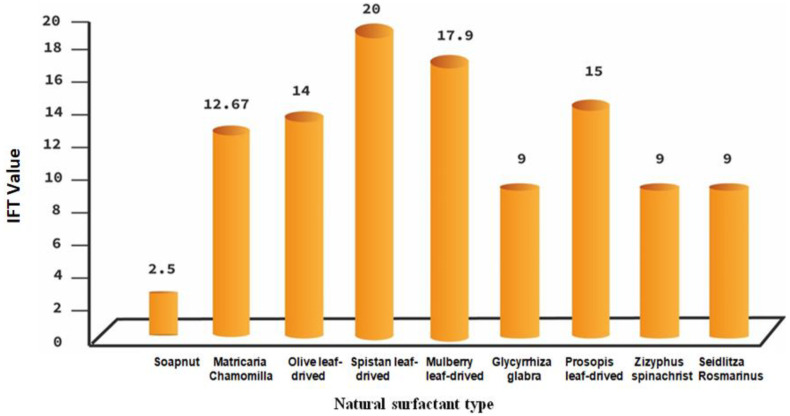
Summary of experimental data for different kinds of natural surfactant.

Natural surfactants mostly derived from the seeds; like chemical surfactants, natural surfactants may be ionic, polymeric, nonionic or amphoteric (Table 2). The critical micelle concentration (CMC) value of synthesized natural surfactant ranges between 9–10 mM, yielding an IFT between 0.075 to 0.125 mNm^−1^. Natural surfactant can also reduce the contact angle efficiently besides IFT reduction. Several extraction methods are explained for synthesizing natural surfactant, but the main methods were spray dryer, soxhlet extraction, methanolic extraction, and maceration process. Different natural surfactant types versus minimum interfacial tension value are depicted in Figure 2. However, Ref. [72] explored the fact that honeycomb micro-porous structures are effective in separating water from oil, hence it may be very significant in diminishing water–oil IFT.

The previous work results (Table 2) declared that, in addition to the commercial surfactants (Table 1), natural surfactants are effective with decreasing IFT, wettability alteration and adsorption on the solid surface. Nowadays, researchers are focusing on applying this type of surfactant because of some advantages and reasonable features such as cost effectiveness, less toxicity, more stability and effectiveness at high pressure and temperature and more biodegradabilities as compared to commercial surfactants (Table 1). These kinds of surfactants can increase oil recovery by about 5–40% of OIIP.

## 4. Potential of NASP Synergism

**Figure 3:**
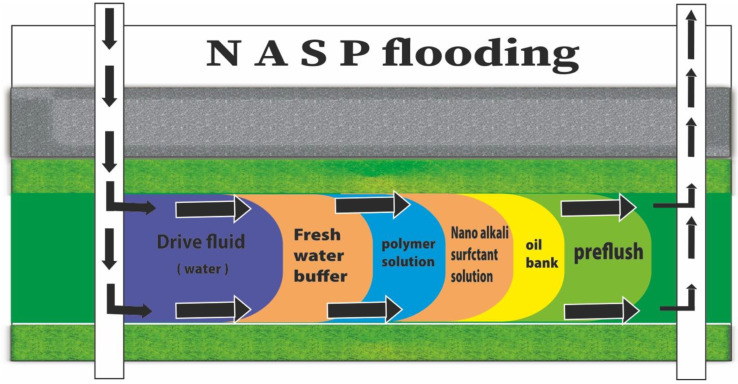
ASP chemical flooding sequence for enhancing oil recovery. ASP EOR injection is shown in sequence at the beginning, the preflush is injected then followed by oil bank; after that, nano-alkaline-surfactant is injected to decrease the IFT followed by a polymer to control the mobility via increasing the viscosity; finally, water drive is used to push the solutions.

To increase the potential of EOR, the optimum chemical solution is achieved at large injection volumes through injecting alkaline, polymer and surfactant (Figure 3). Oil improvement through ASP flooding has been reported previously. The principle of this method is the reaction between alkali and organic to create petroleum soaps. These petroleum soaps will interact with surfactants to reduce the IFT to minimum value (10^−4^ mN/m). In addition, the polymer is used to reduce the viscosity ratio of oil/water interface. Reduction of IFT and oil viscosity significantly improve vertical and displacement efficiency. ASP flooding is used for heavy oil reservoirs [25]. Recently, nanoparticle is mixed with the chemicals above to reduce the cost of these chemicals, modify the wettability, minimize oil-water IFT and boost oil recovery efficiency. Table 3 examines the types of process efficiencies for each of the methods used in the article, and predicts the amount of efficiency by NASP:

(2) $$\mathrm{Ero}\;=\;E_{\mathrm{do}}\;E_{a}\;E_{v}\cdot \frac{So\;Vp}{Bo}$$

where E_do_ is the microscopic displacement efficiency increased by using the optimum surfactant and alkaline type and concentration, *Vp* is the permeability variation and *So* is the oil saturation. E_a_ and E_v_ are displacement efficiencies of areal and vertical, developed by using the appropriate polymer.

## 5. NASP Prediction Technical Characteristics

**Figure 4:**
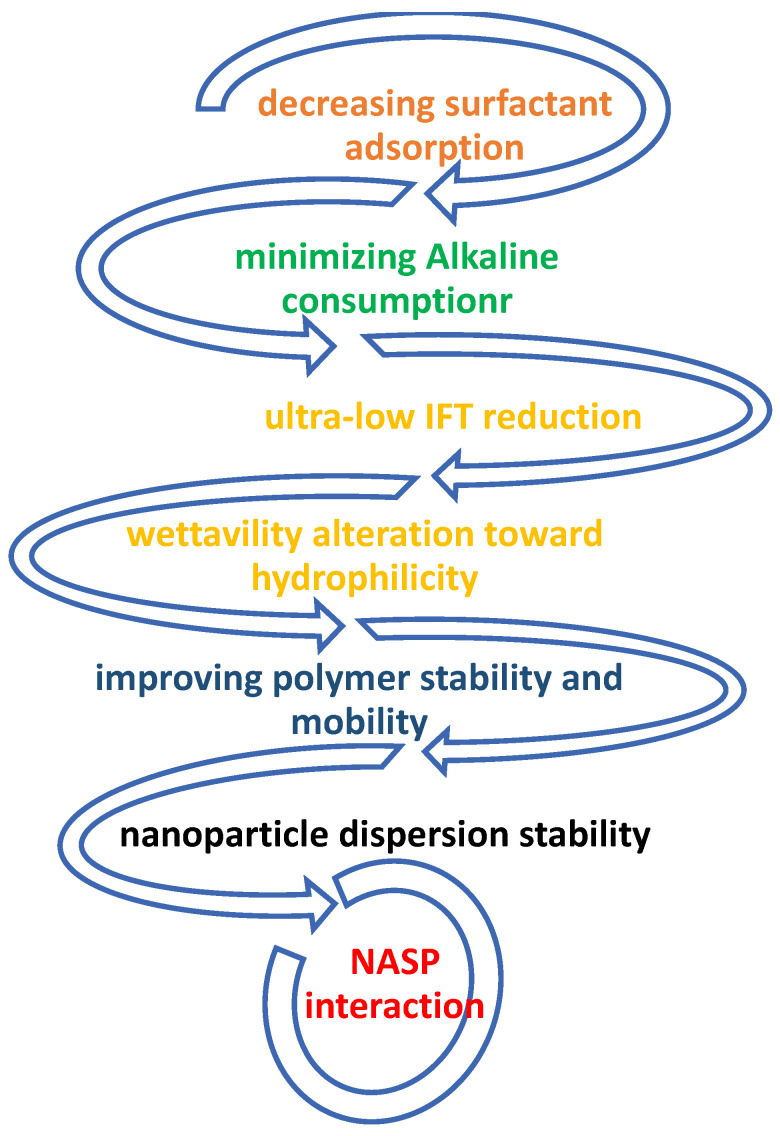
NASP interaction improved mechanisms.

The NASP synergism limits the polymer adsorption and high alkali consumption [82]. Figure 4 illustrates the main reasons for NASP interaction. The predicted technical properties of NASP EOR compared to single element flooding are summarized as follows:

▪ The amount of surfactant is significantly lowered in NASP system;
▪ Strong or a weak base alkali is used in the ASP synergy system;
▪ NASP significantly increases oil recovery since it has physical and chemical (dual) effects;
▪ It is forecasted that, when the four-element composites (N, A, S, and P) are used together, the IFT rapidly decreases to 0.001 or lower.

## 6. Screening the Reservoir Rock Properties

Screening criteria can determine the suitable EOR process for the target reservoir rocks and control the cost issue. Sheng [60] summarized the significant parameters of ASP process, EOR, permeability, clay contents, reservoir temperature, pressure, divalent contents, formation water salinity and oil viscosity.

Due to high anionic surfactant adsorption on carbonate, nearly all the chemical (CEOR) processes were conducted on sandstone reservoir rocks. The presence of anhydrate mineral in carbonate formations caused severe alkaline consumption [83]. On the other hand, clays in sandstone reservoirs caused surfactant adsorption. Thus, clay percentage should be lowered for effective and successful ASP flooding. The permeability is another criterion and very critical to polymer injection in the ASP project since a polymer is not able to flow through tight or low permeable reservoirs.

For alkali, the crude oil composition is a very critical point, while, for polymers, it is not significant [83]. The viscosity of oil should be >35 cP for AS projects. The oil viscosity in Chinese fields projects is from 10–70 cP. According to some authors, it is preferred to apply polymer EOR in reservoirs with viscosity of 2000 cP [84].

ASP projects are more convenient in low salinity reservoirs 10,000 ppm of total salinity [85]. The preferable reservoir temperature is 93 °C for ASP, while the average reservoir temperature for AS field projects was 27 °C, even up to 80 °C, was documented [85]. Recently, scholars have been thinking about using optimum chemicals, in particular polymers, to withstand high salinity and temperature [85].

## 7. ASP/EOR Process Challenges

Even though a chemical ASP process is the most effective chemical process for decreasing water cut and oil enhancement, tight oil produced in water emulsion creates huge problems [86]. In addition, several problems and limitations are associated with offshore ASP/EOR applications [87]. Large chemical volumes that are transported to remote sites, and less space availability, lead to difficulty in operation [86]. Extra treatment is needed for the produced fluid containing alkaline, polymer and surfactant. This tight emulsion formation leads to difficulty and limitation in the separation process [86]. Literature revealed that this chemical process works more efficiently in low salinity water reservoirs [87]. Nevertheless, the source of water injection is from the seawater; therefore, alternative chemicals may be needed. Divalent cations in the system are the main source of scaling.

### 7.1. Operational Difficulties

Like any CEOR injection, ASP are associated with many operational problems such as corrosion, scaling, pump failures, polymer degradation, and low injectivity [82]. In addition, this process is complex in design and needs water and oil analyzing. Moreover, due to a large volume of chemicals, the cost of this process should be analyzed effectively. Finally, this EOR flooding type is not favorable for hot reservoirs or those with saline water [82].

#### 7.1.1. Scaling Issues during ASP Flooding

Calcium and magnesium reaction with the injected alkali leads to a scaling issue. This effect is regarded as one of the ASP limitations since this reaction leads to extreme surfactant precipitation and alkali consumption [88,89]. Several publications reported scaling issues during ASP injection into the reservoir [90,91,92,93]. Scales may originate from the alkalis and carbonate mineral’s reaction. According to the literature, silicate scale formation is a very sophisticated mechanism because the problems associated with silicate are poorly understood. In Chinese oil fields, scaling issues have been observed and reported [93].

#### 7.1.2. Surfactant Precipitation

Divalent cationic existences in hard brines cause surfactant precipitation as illustrated in Equation (3):

2R^−^ + M^2+^ → MR_2_ (3)

where MR_2_ is the surfactant divalent cation, and R the anionic surfactant. Different factors like temperature, alcohol and salt concentration are responsible for anionic surfactants’ precipitation [94]. In most of the cases, the presence of oil reduces surfactant precipitation efficiently since oil competes for surfactant. Ethoxylate (EO) helps surfactant to resist divalent cations. At lower hardness, monovalent cation is formed from the reaction of multivalent cation with the anionic surfactant [94].

## 8. Prospects and Future Developments of ASP/CEOR

Based on this review paper, the following conclusions and recommendations can be proposed for this study:

▪ ASP limitations could be due to alkaline since alkaline reduces polymer viscosity. Thus, a big question is: can SP work more effectively than ASP?
▪ Due to the carbonate rock complexity, most of the nano-EOR flooding has to be performed on sandstone rocks. Further studies should be implemented for understanding the effect of oil recovery on carbonate rocks;
▪ More sophisticated and advanced tools should be used to accurately examine the role of NASP in changing the wettability and IFT;
▪ Due to the lack of economic data in the research papers, more economic study should be implemented to evaluate the economic performance of NASP in accelerating oil recovery;
▪ HS and HT could limit NASP to work effectively in maximizing oil recovery. This is why a more effective nano, surfactant and polymer should be developed to limit this issue.

More research is needed to evaluate the performance of NASP in sandstone and carbonate reservoirs.

## 9. NASP Performance Anticipation in Changing the Wettability and IFT

**Figure 5:**
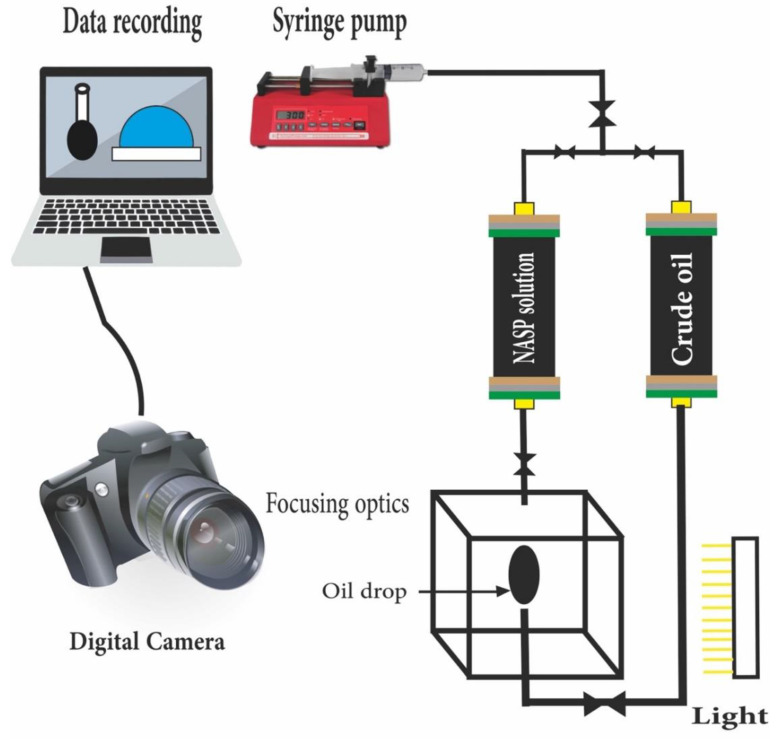
IFT and contact angle measurement by using pendant drop (oil drop).

After reviewing and evaluating ASP lab and field projects, it is forecasted that NASP could modify wettability and IFT effectively more than any other previous chemical methods due to the synergism effect of ASN (Alkaline Surfactant Nano), and it may stabilize the polymer solution excellently due to the NP synergism effect. Based on the above points, ultimate oil recovery could be guaranteed by implementing NASP. IFT and contact angle are the main parameters of any EOR type since it is related to the capillary number modification. Due to accuracy and ease of use, pendant drop is considered as one of the main methods to calculate IFT and contact angle. IFT study will be implemented by introducing a drop into a bulk phase under the desired P and T. For contact angle measurement, the drop is completed on a plane solid sample. Pictures captured by a digital camera connected to a computer show the shape of the drop and allow for solving the Laplace equation contact angle and IFT calculation. Figure 5 illustrates IFT and contact angle measurement by using pendant drop.

## 10. Core Flooding

**Figure 6:**
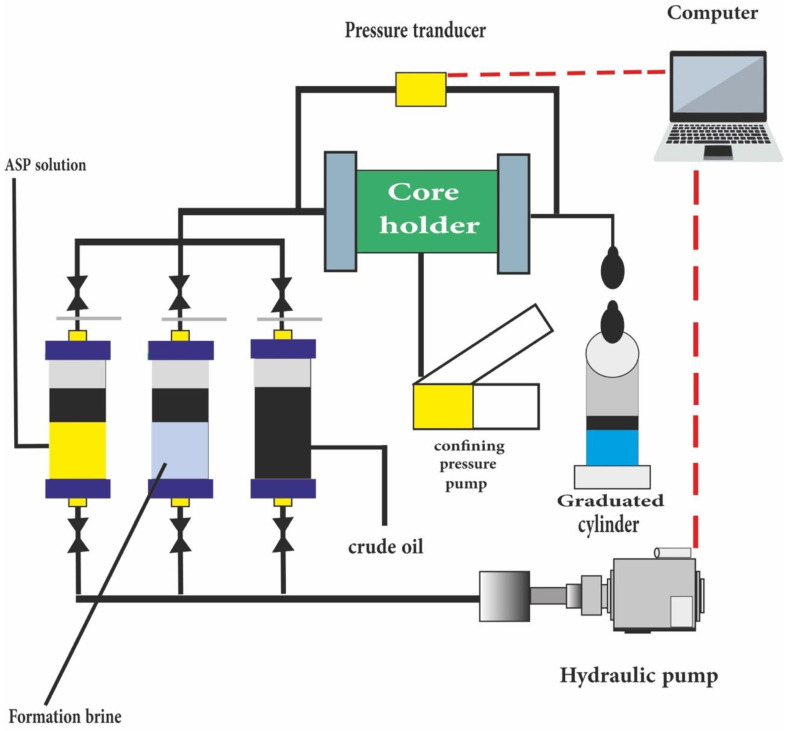
Core–flood procedure.

For recovery measurement, a dynamic test will be prepared by core-flooding. Different core plugs are used with injecting best chemical, nanofluid and hybrid nanochemical solutions under the reservoir condition. For this purpose, a core sample should undergo several procedures to be prepared and aged. After aging, the brine solution is injected, followed by the injection of a hybrid Nano-ASP solution to study oil recovery from the carbonate rock. Then, the values of oil production are recorded vs. time. Finally, oil recovery is plotted vs. pore volume. This review paper forecasted that oil recovery by NASP could be more than 25% due to the improved mechanisms that have been discussed in the other sections. Figure 6 illustrates NASP core–flood procedure.

## 11. Future Design, Materials and Features of NASP Process

### 11.1. Nano-EOR

**Figure 7:**
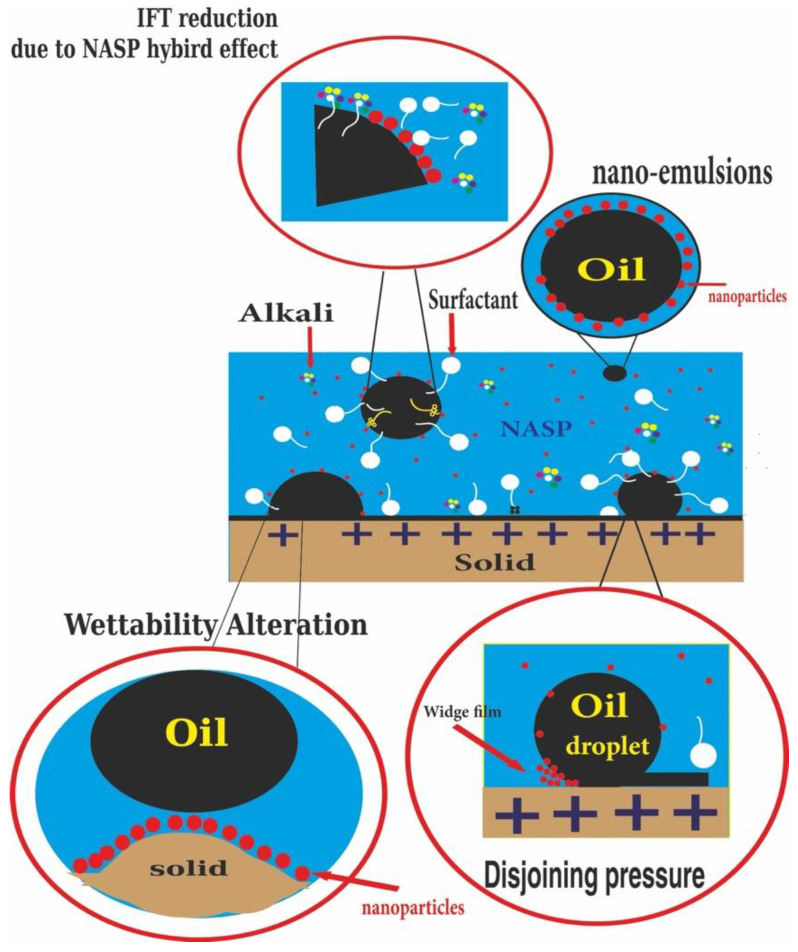
IFT reduction and wettability alteration by nanoparticles.

Nanoparticles are wide classes of material substances, with sizes between 1–100 nm. Nowadays, the oil and gas industry has attracted much consideration on applications of nanoparticles (NPs). NPs have become widely used due to having unique optical, magnetic and electric features. Mixing nanoparticles with other substance phases is called nanocomposites (NCs). NPs within different dispersion media can be easily transported through the porous media and reach the oil bank due to their smaller sizes, less than micron-sized rock pores. Consequently, NCs can be used to decrease ASP adsorption with the rock surface, altering the wettability of the rock, reducing the interfacial tension (IFT) and improving oil displacement and recovery (Figure 7). The surface area-to-volume ratio of nanoparticles is too large, where a small concentration of them is needed to induce EOR injection fluids. Combining nanoparticles with the natural polymers develop polymeric nanofluids, thus the resulted NPs would have a better stability, mobility of injection fluids and sweep efficiency.

Figure 7 highlights that the nanoparticles can alter the wettability and reduce oil water IFT. The mechanism of wettability alteration by nanoparticles is to build a wedge film on the oil droplet and the rock surface. The nano size particles re-arrange themselves between the rock and oil droplet, leading to oil separation and thus altering the wettability from hydrophobicity to hydrophilicity, and decreasing the excessive surfactant adsorption. Moreover, disjoining pressure is regarded as one of the nanoparticle mechanisms in EOR since it responsible for changing the oil wet surface to water wet. This mechanism is highly affected by nanoparticle type and concentration. Different nanoparticle types play different roles in the EOR mechanism process (Table 4).

### 11.2. Summary of Nano (NASP) EOR Flooding

Nanoparticles are very effective in changing the wettability and contact angle. Recently, nanoparticles were mixed with surfactant, polymer and SP to further improve oil recovery. The shape, size, dispersion media, nature and concentraion of nanoparticle will govern the most suitable and effective nanoparticles for achieving the best EOR mechanisms (Table 4). The main advantages of using nanoparticles are their large surface areas with spherical nanoparticles being more effective than any other nanoparticle shapes.

The main parameters that control the nanoparticle shape is the temperature, pH and time. In addition, nanoparticles play an astonishing role in improving the oil recovery since smaller nanoparticle size leads to lower IFT and contact angle (Figure 7). Furthermore, lab works hypothesized that optimum concentration should be guaranteed for any EOR application; otherwise, nanoparticles will agglomerate, leading to lower recovery efficiency. Regarding the dispersion media, our review paper finds out that different oil recovery is accomplished through dispersing nanoparticles into different dispersion media. However, Rajabi et al. used the wettability modifier: nanoparticles, surfactant and alkaline, but the highest percent of oil recovery was obtained by nano-surfactant rather than by adding alkaline [95]. The latter did not cause any positive impact on EOR. Furthermore, the oil improvement, using different kinds of nanoparticles, nano-polymers, nano-surfactants and nano-surfactant-polymers, efficiently decreases contact angles and IFT (Table 5), despite the fact that heterogeneity of sedimentary rocks could influence the quantity of oil improvement. The sedimentary rocks, especially carbonate rocks, characterized intensive lithological changes along micrometer-sized scales in both subsurface [96] and near-surface conditions [97]. This heterogeneity in enhanced oil recovery is the main cause behind the failure of oil recovery in the field scale. Therefore, the new model or design (NASP) for future experimental work should be achieved with detailed work on reservoir characteristics, including mineralogy and, of course, by detailed observations (both optical microscope and SEM).

This work systematically investigates the potential of NASP suspension for enhanced oil recovery (EOR) in carbonate and sandstone reservoirs. Using NASP (Nano/Alkaline/Surfactant/Polymer) could be proved successfully in future work due to its ability to improve displacement and sweep efficiency.

In the case of NASP, various mechanisms will be activated, and the interaction of them will be important, which we will describe separately to better understand the issue:

- The capability of the nano-polymer suspensions for improving the oil recovery by the following mechanisms:
  1. Wettability alteration was explored using contact angle measurement; increasing temperature and adding salt to polymeric solutions caused a reduction in shear viscosity, and the addition of NPs to the solutions could relatively recover the viscosity;
  2. The presence of polymers in the nanofluids improved dispersion stability of NPs;
  3. The nano-polymer suspensions could improve the ability of the NPs for wettability alteration and faster equilibrium states obtained than the polymer-free nanofluids.
- The performance of the nano-surfactant solutions for improving the oil recovery by the following mechanisms:
  1. The adsorption process of these substances is one of the important methods to increase the oil recovery factor from oil reservoirs by wettability alteration;
  2. The results of the IFT experiments of these materials showed that surfactant nanofluid solutions could significantly reduce the IFT value between the oil and water system.
- Alkaline can activate the following mechanisms:
  1. Interfacial tension reduction;
  2. Wettability alteration;
  3. Control of adsorption of ions;
  4. Improving the emulsion stability;
  5. Inhibitor of clay swelling.

Suitable experimental design is critical for using nanoparticles with chemical EOR. This paper for the first time will investigate a detailed schematic diagram for using nanoparticles in hybrid with ASP for future perspectives.

**Figure 8:**
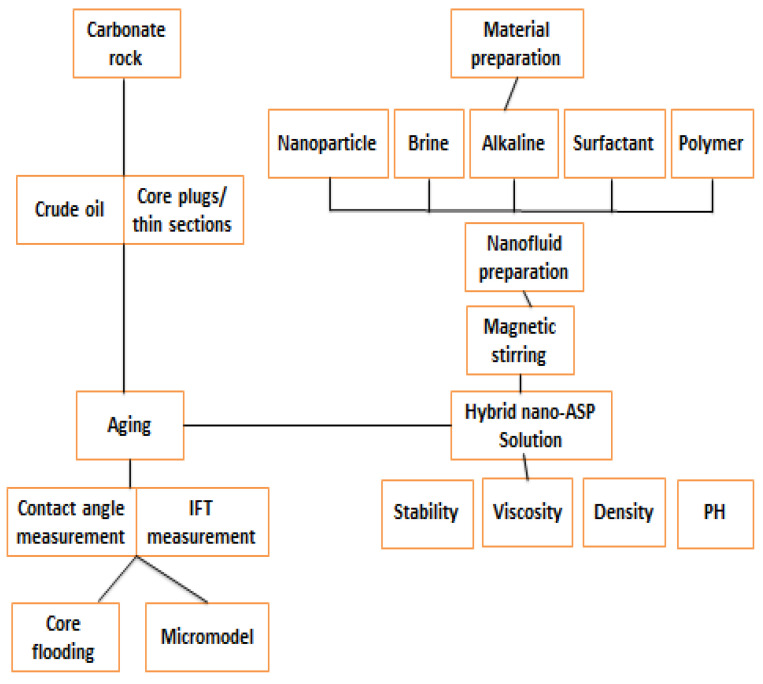
Future experimental work. The figure forecasted NASP experimental work methodology. The latter is subdivided into several sections: material preparation, carbonate rock preparation and EOR static tests (IFT and contact angle) and Dunamis tests (core flooding and micromodel).

## 12. Conclusions

1. To sum up, CEOR was applied to greatly increase the ultimate oil recovery by wettability, IFT and mobility modification. This paper will add a new insight integrating nano-alkaline, polymer and surfactant flooding for the first time by addressing the main mechanism of each one. The main conclusions of this paper are as follows:
2. ASP limitations could be due to alkaline since alkaline reduces polymer viscosity;
3. Due to nano, surfactant, polymer, and alkaline synergy effects, most of the EOR mechanisms are greatly improved, leading to higher oil recovery as compared to using each component alone;
4. The objective behind using NASP in hybrid is to modify wettability, IFT and mobility ratios, which are regarded as the main EOR mechanisms;
5. NASP type and concentration play a major role in changing wettability and reducing IFT to a minimum level;
6. For checking the mobility of chemical EOR, the micromodel is used to find the fluid flow distribution;
7. Nanoparticle type and size play a major role in changing wettability and reducing IFT to the minimum level;
8. Future recommendations by utilizing NASP will probably be a new finding to understand the details about the EOR system in both micro- and -macroscale settings;
9. This review paper highlights the fact that natural surfactants are less costly, biodegradable, available, less toxic, more stable, and environmentally friendly, and it can reduce the IFT to an ultra-low value.
10. NASP could effectively boost the oil recovery by more than 25% due to the synergism effect.

## Figures and Tables

**Table 1:** Summary of previous experimental and field ASP works alone and in synergy.

CEOR Type	Ref.	Chemical Name		Conc.	Porosity %	Permeability (md)	Lithology
**Alkaline**	[12]	Na_2_CO_3_		1.0	NA.	405–608	Sandstone
		NaOH		1.0			
		Na_4_Si0_4_		0.5			
	[13]	Na_2_CO_3_		0.85	25	70	Sandstone
	[2]	NaOH		0.5	NA.	1580	Sandstone
	[14]	NaOH		0.5	33.59	6000	Sandstone
	[15]	NaOH		0.2	NA	NA	Carbonate
	[2]	Na_2_CO_3_		NA	35.5	3200	Sandstone
	[16]	NaOH		0.5	38.7	NA	Sandstone
	[17]	NaOH		0.2	NA	NA	Sandstone
	[18]	NaOH		NA	15	119	Sandstone
	[19]	NaOH		4.85	20.6	25	Sandstone
	[20]	NaOH		0.15	16	NA	Sandstone
	[21]	NaOH		0.2	30	495–320	Sandstone
	[22]	NaOH		0.5	NA	2110	Sandstone
	[23]	NaOH		1.0	NA	NA	sandstone
	[24]	NaOH		2.0	19.7	93.64	Sandstone Sandstone
		Na_2_CO_3_		2.0	19.6	176.25	
	[25]	Na_2_C0_3_		1.20	35	3850	Sandstone
**Surfactant**	[26]	Cationic C_12_TAB		NA	45–50	2–5	Carbonate
	[27]	SDS		1000	52.2	250	Carbonate
	[25]	OPIO and CY1		NA	35	3850	Sandstone
	[28]	Anionic surfactant		NA	29.06	19.72	Carbonate
	[29]	Nonionic surfactants		NA	NA	20.89	Carbonate
	[30]	Anionic surfactant		NA	3–5	NA	Carbonate
	[31]	nonionic ethoxy alcohol		3000–4000	3–5	NA	Carbonate
	[32]	Anionic (GAC) surfactants		NA	NA	NA	Carbonate
	[33]	Nonionic (POA)		750	15.4	56	Carbonate
	[34]	Nonionic ethoxy alcohol		50–3500	NA	NA	Carbonate
	[16]	SDS		1000	38.6	212	Sandstone
	[35]	SDBS		250	37	284	Sandstone
	[36]	SLPS		1000	38	NA	Sandstone
	[37]	SDS		3000	NA	NA	Glass bed
	[38]	XD		1000	NA	NA	Sandstone
	[39]	SDBS		2000	25	NA	Sandstone
	[13]	SDS		NA	23–29	50–94	Sandstone
**Polymer**	[12]	PAM		3200	NA	405 to 608	Sandstone
		Xanthan gum		1540	NA	405 to 608	Sandstone
	[36]	(HPAM)		1000	37.5	3780	Sandstone
	[40]	(Gum Arabic/Poly acrid		NA	NA	NA	Sandstone
	[41]	HPAM		1100	21.6	420	Sandstone
	[42]	TVP		2000	NA	NA	Sandstone
	[43]	HPAM		1800	NA	NA	Sandstone
	[44]	PAM		500	>39	100–60	Sandstone
	[16]	PHPAM		1500	37.3	218	Sandstone
	[45]	HPAM		1200	15.2	23.34	Sandstone
**Alkaline Surfactant**	[46]	Xylene + NaOH		5000 + 10,000	NA	NA	Carbonate
	[47]	IOS + Na_2_CO_3_		(200–10,000) + 5000	37	2400	Micromodel
	[48]	Na_2_CO_3_ + alkyl ether sulfates		1500 + 50	NA	NA	NA
	[38]	Na_2_CO_3_ + XD		10,000 + 1000	NA	NA	Sandstone
		Na_2_CO_3_ + SDS		10,000 + 10,000	NA	NA	Sandstone
	[49]	(IOS) + NaOH + Na_4_Si0_4_		NA	NA	NA	Sandstone
	[50]	NaOH + SLPS		3000 + 300	44.7	2131	Sandstone
		NaOH + SLPS		8000 + 1000	43.50	1994	
		NaOH + SLPS		10,000 + 1000	44.21	2016	
	[13]	SDS + Na_2_CO_3_		1000 + 8500	25	70	Sandstone
	[51]	Na_2_CO_3_ + sodium alkane sulfonate		(1000–15,000) + 1000	41–45	790–19,220	Sandstone
**Alkaline polymer**	[52]	Na_2_CO_3_ + anionic PAM		NA	15.5	21	Sandstone
	[53]	Na_2_CO_3_ + Pusher 1000E		8000 + 600	29	1400	Sandstone
	[54]	NaOH + Alcoflood 1275A		10,000 + 1000	20	200	Sandstone
	[55]	NaOH + HPAM		10,000 + 1000	38.92	2350	Sandstone
		Ethylenediamine + HPAM		10,000 + 1000	39.41	2230	
		Na_2_CO_3_ + HPAM		10,000 + 1000	40.33	2420	
	[56]	Na_2_CO_3_ + anionic PAM		NA	15.5	21	Carbonate
	[57]	(NaOH + Na_2_CO_3_) + HPAM		(4000 + 2000) + 250	34.43	4800	Sandstone
	[57]	HPAM + (NaOH + Na_2_CO_3_)		1000 + (1000 + 2000)	35.25	6400	Sandstone
	[52]	Na_2_CO_3_ + Alcoflood 1175		10,000 + 800	29	1400	Sandstone
	[58]	Na_2_CO_3_ + PAM		10,000 + 1500	31	840.9	Sandstone
	[25]	Na_2_C0_3_ + OP-10		1200 + 10,000	35	3850	Sandstone
	[59]	Na_2_CO_3_ + HPAM		20,000 + 1000	27.6	2063	Carbonate
**Surfactant polymer**	[60]	alkyl ether sulfates + Witco petroleum sulfonate		1000 + 1000	12	5.9	Carbonate
	[61]	Amphoteric + PAM		2500 + 1400	29.1	3442	Sandstone
	[62]	PAM + SDS		1000 + 2200	21	66	Sandstone
	[16]	SDS + PHPAM		1000 + 2000	36.8	1224	Sandstone
	[63]	bio-surfactant and biopolymer		1001 + 5000	17	400	Sandstone
	[61]	PS + PAM		2000 + 2000	21	115	Sandstone
	[39]	SDBS + HPAM		2000 + 2000	25	NA	Sandstone
	[64]	PAM + SDS		2800 + 1000	NA	NA	Glass micromodel
		PAM + SDS		2800 + 2000			
		PAM + SDS		2800 + 3000			
	[61]	(Amphoteric +anionic) + PAM		1200 + 1500	15	110	Sandstone
	[36]	HPAM + SDS		1000 + 1000	38	1410	Sandstone
	[45]	anionic surfactant + HPAM		1200 + 1200	15.2	23.34	Sandstone
	[43]	SLPS + HPAM		4000 + 1800	NA	1500	Sandstone
	[65]	KPS + HPAM		3000 + 115	14.7	5.08	Sandstone
	[43]	SLPS + (HPAM)		4000 + 1800	NA	1500	Sandstone
**Alkaline surfactant polymer (ASP)**	[60]	NaOH + SDS + PHPAM		5000 + 1000 + 2500	NA	NA	Sandstone
		NaOH + SDS + PHPAM		5000 + 2000 + 2500			
		NaOH + SDS + PHPAM		5000 + 3000 + 2500			
	[66]	Amphoteric Petrostep B-100 + Pusher 700E + Na_2_CO_3_		4000 + 1200 + 20,000	8–43	1–600	Carbonate
	[67]	Na_2_CO_3_ + SDS+ PAM		10,000 + 1000 + 800	NA	NA	Sandstone
	[16]	NaOH + SDS + PHPAM		5000 + 1000 + 1500	38.7	NA	Sandstone
		NaOH + SDS + PHPAM		7000 + 1000 + 1500			
		NaOH + SDS + PHPAM		10,000 + 1000 + 1500			
	[16]	NaOH + SDS + PHPAM		5000 + 1000 + 1500	38.7	NA	Sandstone
		NaOH + SDS + PHPAM		5000 + 1000 + 2000			
		NaOH + SDS + PHPAM		5000 + 1000 + 2500			
	[68]	Na_2_CO_3_ + Petrostep B-100 + Alcoflood1175A		12500 + 1000 + 1475	18	845	Carbonate
	[69]	Diethylene glycol butyl ether + alcoflood-2545 + NaBO_2_		3000 + 10,000 + 10,000	17.7	239	Sandstone
	[70]	HAPAM + NaOH + heavy alkylbenzene sulfonate		1000 + 1200 + 3000	NA	252	Sandstone
	[71]	Na_2_CO_3_ + (anionic BES and lignosulfonate PS) + PAM		12,000 + 3000 + 1700	NA	NA	Sandstone
**CEOR** **Type**	**Ref.**	**Work Type**	**Oil Improvement %**	**Remark**			
**Alkaline**	[12]	Experimental	17.2	Na_2_CO_3_ is more effective for oil increment			
			9.42				
			8.91				
	[13]	Experimental	4.4	Due to soap formation, Interfacial tension between oleic and aqueous phase reduced			
	[2]	Experimental	12.4	Low salinity leads to O/W emulsions if the salinity is above 0.7 W/O emulsions happen			
	[14]	Experimental	13.33	IFT reduction due to soap formation improves oil recovery			
	[15]	Experimental	NA	Alkaline flooding is more applicable for medium crude oil as compared to light crude oil due to a higher ratio of soap formation in medium crude oil			
	[2]	Experimental	14	Alkaline is also applicable and can accelerate oil recovery in horizontal wells			
	[16]	Experimental	13.88	Strong base (NaOH) alkaline injection enhanced oil recovery			
	[17]	Experimental	NA	Higher oil recovery due to in situ. Emulsion formation			
	[18]	Experimental	2	additional oil guaranteed by changing the wettability			
	[19]	Field	6–8	the amount of IFT reduction determines the success of alkali job			
	[20]	Field	2	Changing in rock surface wettability directly affects oil recovery			
	[21]	Field	5 to 7	Formation of the emulsion by alkaline improves volumetric sweep efficiency			
	[22]	Experimental	12.9	The oil displacement experiment proved that oil recovery is enhanced by using alkaline injection			
	[23]	Experimental	<1	Orthosilicate was very successful at stopping water channeling and increasing oil recovery			
	[24]	Experimental	2.52 3.67	NaOH is more effective than Na_2_CO_3_			
	[25]	Field	9.13	Ultra-low IFT after alkaline flooding			
**Surfactant**	[26]	Experimental	20	changing rock surface wettability due to the sulfate that is present in the injection fluid			
	[27]	Experimental	9	Oil recovery affected by the type and concentration of the surfactant used in the formation			
	[25]	Field	11.64	Higher amount of IFT reduction leads to more oil recovery			
	[28]	Experimental	30	Optimum surfactant concentration is related with brine salinity			
	[29]	Field	NA.	About 58,000 bbl of oil is produced after using Nonionic surfactants over only three months			
	[30]	Experimental	NA	The performance of anionic surfactants was more effective than nonionic surfactants			
	[31]	Experimental	15.0	to optimize surfactant performance injection rate, conc. and volume are the important parameters			
	[32]	Experimental	NA	IFT significantly diminished			
	[33]	Experimental	10.4	Nonionic surfactant outperformed cationic surfactant			
	[34]	Experimental	NA	surfactants decreased IFT and changed the contact angle			
	[16]	Experimental	17.96	IFT decreased marginally			
	[35]	Experimental	2.1	Due to surfactant degradation, the oil recovery was low			
	[36]	Experimental	4	Increase in capillary number yields more oil recovery			
	[37]	Experimental	NA	IFT reduced from 19.59 to 2.82 mN/m			
	[38]	Experimental	11.5	At lower concentration, a novel XD yielded a good oil recovery that can be compared with SDS			
	[39]	Experimental	4.6	Compared to SP flooding, oil recovery by using surfactant flooding was reduced			
	[13]	Experimental	7.1	Adsorption phenomena indicated that SDS was a suitable choice for sandstone formation			
**Polymer**	[12]	Experimental	11 10.5	Polymer type selection is critical			
	[36]	Experimental	10.7	Increasing the viscosity of water by HPAM improves vertical sweep efficiency			
	[40]	Experimental	5.2	Core flood test indicates that this type of polymer is less effective than other polymer types due to less oil improvement			
	[41]	Field	9.8	Higher molecular weight improves thermal stability			
	[42]	Experimental	13.5	Temperature affects polymer performance			
	[43]	Experimental	6.3	PPG is more effective in higher and lower permeability zones compared to conventional polymer (HPAM)			
			13.4				
	[44]	Field	7	Earlier injection of polymer is more profitable			
	[16]	Experimental	16.12	High viscosity of polymer leads to an increase in macroscopic displacement			
	[45]	Experimental	8.80	From the results, it can be indicated that polymer is used mostly to reach to the unrecoverable oil zones			
**Alkaline surfactant**	[46]	Field	10–15	Alkaline-surfactant flooding offers a potential scheme to recover part of the high residual oil that was not recovered by waterfront			
	[47]	Experimental	NA	Emulsification of heavy oil by AS was effective			
	[48]	Experimental	NA	For mobilizing heavy oil, AS flooding is a very suitable choice			
	[38]	Experimental	14.58	In situ soap by alkali and surfactant reduces IFT significantly			
			10.42				
	[49]	Field	NA	Alkaline is not able to mobilize oil alone, when surfactant added IFT reaches minimum value and oil easily mobilized			
	[50]	Experimental	12.10	Surfactant and soap (in situ) surfactant formation efficiently reduces IFT			
			15.80				
			18.63				
	[13]	Experimental	18	As shown in adsorption phenomena, alkali plays a major role in reducing surfactant adsorption			
	[51]	Experimental	10.5	Surfactant reduces the alkaline consumption			
**Alkaline polymer**	[52]	Field	NA	AP synergy effect was efficient for improving EOR mechanisms			
	[53]	Field	21.1	AP was sufficient to improve oil recovery			
	[54]	Field	17	Binary system of A and K performance is more significant than using each of the chemicals alone			
	[55]	Experimental	21.02	In AP flooding, alkaline selection plays a critical role in oil recovery improvement			
			25.21				
			18.12				
	[56]	Field	26.4	Soap formation by Na_2_CO_3_ and viscosity improvement by anionic polymer yielded higher recovery			
	[57]	Experimental	18.58	Conc. of polymer influences oil recovery			
			27.60				
	[57]	Experimental	16.56	Conc. of Alkaline influences oil recovery			
			27.39				
	[52]	Field	21.1	67% OOIP was recovered by AP			
	[58]	Experimental	22.8	Polymer solution should be injected at a good speed			
	[25]	Field	18.12	alkali cannot to the oil region without polymer			
	[59]	Experimental	1.98	Mobility control improved			
**Surfactant Polymer (SP)**	[60]	Experimental	12.0	Using two surfactants was more effective			
	[61]	Field	16.3	Using surfactant with polymer yield extra oil recovery			
	[62]	Experimental	17.25	Temperature and initial oil saturation affects oil recovery			
	[16]	Experimental	20.99	Better mobility control is obtained by using polymer with surfactant			
	[63]	Experimental	15.94	The binary system demonstrated high interfacial activity with IFT min below 0.01 mN/m			
	[61]	Field	13.8	Synergism of polymer and surfactant further improves oil recovery			
	[39]	Experimental	20	Oil recovery after using dual chemicals (S and P) was higher than the total oil that is produced by using S and P alone			
	[64]	Experimental	41	CMC of SDS is 0.21 means that higher concentrations of CMC have a marginally effect on oil recovery			
			41.4				
			42				
	[61]	Field	14.5	Polymer and surfactant synergism developed by choosing the optimum conc. of each			
	[36]	Experimental	13.7	Without polymer injection surfactant cannot go through unsweep zones			
	[45]	Experimental	11.29	Anionic surfactant for sandstone reservoir is very effective			
	[43]	Experimental	13.6	Polymer and surfactant synergistic yields higher oil displacement			
	[65]	Experimental	23.96	Polymer controls mobility control and surfactant reduces IFT			
	[43]	Experimental	22.4	SLPS improves displacement efficiency and (HPAM + PPG) improves sweep efficiency			
**Alkaline surfactant Polymer (ASP)**	[60]	Experimental	23.69	Increase in surfactant conc. leads to oil recovery enhancement			
			27.18				
			28.72				
	[66]	Experimental	45	An alkaline surfactant polymer formulation was substantially better in recovering oil than surfactant or polymer surfactant			
	[67]	Experimental	7.4	A, S and P synergism yielded higher oil recovery. Alkaline reduces surfactant adsorption. Surfactant reduces alkaline consumption and polymer increases the viscosity of water. These three functions play a great role in recovery enhancement			
	[16]	Experimental	23.69	Effect of different alkaline concentration in ASP slug yields different oil recovery, indicating that optimum concentration of alkaline should be guaranteed			
			24.08				
			24.91				
	[16]	Experimental	23.69	Optimum concentration of polymer is required during ASP injection for higher oil recovery			
			23.5				
			24.2				
	[68]	Field	28.1	ASP synergy effect makes the process efficient			
	[69]	Field	10–28	Pore scale displacement efficiency improved due to synergy of three chemicals			
	[70]	Field	>25	NaOH and heavy alkylbenzene sulfonate reduces IFT dramatically and polymer pushes the heavy oil			
	[71]	Experimental	15.5	Present the co-surfactant in the ASP slug is critical in releasing the trapped oil in the porous part of the reservoir rock			

Different kind of ASP flooding in different reservoir characteristics, close up percentage of oil improvement. From this table, it is obvious that ASP is one of the promising EOR applications due to its dual efficiency effect, alkaline and surfactant microscopic efficiency, and polymer progresses volumetric sweep. Reservoir fluid and rock property play a vital role for ASP selection, and, for achieving highest oil recovery, suitable ASP type and concentration should be used. For sandstone formation, the convenient surfactant type was used in ASP is SDS. CTAB is mostly used for carbonate due to its surface charge. Lith = Lithology; P. = Porosity; Pb. = Permeability; Sst = Sandstone Carbo. = Carbonate; Qz = Quartz; SDS = sodium dodecyl sulfate; GAC = Guerbet alkoxy carboxylate; POA = polyoxyethylene alcohol; PAM = Polyacrylamide: HPAM = hydrolized polyacrylamide; C_12_TAB = Dodecyltrimethylammonium (bromide); SDBS = Sodium Dodecylbenzene Sulfonate Surfactant; KPS = Potassium persulfate; XD = xylitoldehydrogenase; TVP = thermo-viscosifying polymer; IOS = internal olefin sulfonate; PS = Pulmonary surfactant; SLPS = surfactant like peptides.

**Table 2:** Summary of natural surfactant types and their properties.

Ref.	Name	CMC	Type	IFT From-to mN/m	Contact Angle From-To	Oil Recovery Improvement %	Properties
[73]	Reetha Extract	2.3	Natural non-ionic	18.6 to 7.02	NA	6.8	Applicability of new surfactant and increase oil recovery from 18.5 to 25.3
[74]	Mulberry leaves extract	2.6	Natural cationic	44 to 17.9	62.5° to 42.5	7	Suitable for carbonate rock
[75]	Matricaria chamomilla extract	0.05	Natural Nonionic	30.63 to 12.53	NA	NA	Good IFT reduction ability
[76]	Cordia Myxa plant	0.06	Natural	33 to 16.24	NA	27	Good adsorption
[77]	Mahua oil	NA	NA	10^−2^	NA	20	Applicable for sandstone reservoir
[78]	Seidlitzia rosmarinus extract	0.08	Cationic	32 to 9	NA	NA	The reduced IFT is not as low for EOR application
[79]	Jatropha oil-based	NA	Nonionic	0.917	NA	25	Good surface activity
[80]	Henna extract	0.02	Cationic	43.9 to 3.05	66 to 37	7	Good wetting ability
[81]	Olive leaf extract	1.95	Natural cationic	36.5 to 14	NA	NA	Good adsorption
[81]	Spistan leaf Extract	2.1	Natural cationic	36.5 to 20.15	NA	NA	good associative and interfacial properties
[81]	Prosopi leaf Extract	2.3	Natural cationic	36.5 to 15.1	NA	NA	Good adsorption

**Table 3:** NASP oil recovery efficiency prediction and efficiency of ASP alone and in synergy.

CEOR Type	Oil Recovery %	Basic Principle
Nano	5–23	Improvement in sweep and displacement
Alkaline	2–5	Improvement in displacement
Polymer	2–10	Improvement in sweep
Surfactant	5–15	Improvement in displacement
SP	5–20	Improvement in sweep and displacement
AP	5–18	Improvement in sweep and displacement
ASP	5–25	Improvement in sweep and displacement
Nano-polymer	4–20	Improvement in sweep and displacement
Nano-surfactant	5–20	Improvement in sweep and displacement
NSP	8–22	Improvement in sweep and displacement
**NASP**	**>25 (predicted by our study)**	**Improvement in sweep and displacement**

**Table 4:** Role of different nanoparticles in EOR mechanisms.

Nanoparticle	Nano-Composites	EOR Mechanisms
SnO_2_, ZrO_2_, Carbon nanoparticles	CTAB + Al_2_O_3_	Wettability alteration by disjoining pressure
SiO_2_	NiO + SiO_2_	Change wettability of oil by disjoining pressure
ZnO	SDS + ZrO_2_	Decreasing the contact to water wet by disjoining pressure
Al_2_O_3_	SiO_2_ + PAM	Wettability alteration by disjoining pressure
Fe, SiO_2_, GO, TiO_2_	ZrO_2_, NiO	Reduce interfacial tension
Al_2_O_3_, CuO, Fe_2_O_3_	SDS + Al_2_O_3_	Reduce the viscosity of crude
MgO	CuO/TiO_2_ + PAM	Optimized permeability

**Table 5:** Summary of nano, nano-surfactant, nano-polymer and nano-SP flooding. It is concluded that a combination of nanoparticle and nanocomposites with conventional CEOR methods improves the synergism effect. As shown in the summary table, nanoparticles are used to support alkaline and surfactant in IFT reduction and wettability modifications (microscopic improvement). In addition, it is used to lower surfactant costs through controlling surfactant adsorption on the rock surface. In the case of polymers, nanoparticles improve the mobility of the polymeric solution, resulting in better oil sweep efficiency and reducing breakthrough time (macroscopic improvement). Lith = Lithology; Sst = Sandstone; Carbo. = Carbonate; Qz = Quartz.

Ref.	Nano Type	Lith.	Contact Angle		IFT		Oil Improvement %	Remark
			Before	After	Before	After		
[98]	SiO_2_	Qz	131	38.82	19.2	17.5	2	Different nanoparticles’ type and size have different performances
	TiO_2_		131	21.64	19.2	NA	11	
	Al_2_O_3_		131	28.6	19.2	12.8	8	
[99]	SiO_2_	Sst	51	30.5	21	20.3	10.1	Due to NP adsorption, the wettability altered from oil to water wet
[100]	SiO_2_	Sst	NA.	NA	NA.	NA.	4.29	Wedge film creation by nanoparticles
[101]	SiO_2_	Sst	122	16	13.62	10.69	23.5	Increase in capillary number due to SIO_2_
[102]	graphene nanosheets	Sst	NA.	NA.	NA.	NA.	6.7–15.2	Size of nanoparticle plays a great role in EOR
[103]	SiO_2_/TiO_2_	Sst	154	23	NA.	NA.	NA.	Spherical shape of nanoparticle improves uniformity
	Al_2_O_3_/TiO_2_		154	24				
[104]	HLP	Sst	135.5	95	26.3	1.75	32.2	NPS yields higher oil recovery without creating any damage to the formation
	NWP		135.5	82	26.3	2.55	28.57	
[105]	NiO/SiO_2_ NCs	Carb.	174	32	28	1.84	NA	NiO/SiO_2_ Nanocomposite responsible for altering the wettability in carbonate rock reservoir
[106]	LHPN	Sst	35	<10	NA.	NA.	1.92	Capillary number improvement leads to oil enhancement
	NWPN		35	0			29.23	
	HLPN		35	NA.			29.01	
[107]	SiO_2_	Sst	12	40	17.5	7	28	Sweep efficiency improved by IFT reduction
[108]	TiO_2_	Carb.	55.3	61.9	17.5	12.5	6.6	Temperature affects oil recovery
	SiO_2_		54.8	57.7	16.7	11.1	2.9	
[109]	γ-Al_2_O_3_	Carb.	119.8	40	NA.	NA.	11.25	γ-Al_2_O_3_ plays the main role in altering the wettability from oil to water wet
[110]	Al_2_O_3_	Sst	53.68	28.6	NA.	NA.	NA.	As a result of nanoparticle deposition, rock surface altered to water wet
	TiO_2_		53.68	21.6				
	SiO_2_		53.68	38.8				
[111]	Al_2_O_3_	Sst	NA.	NA.	NA.	NA.	12.5	For guaranteeing optimum oil recovery design, engineers should select the effective nanoparticle type and size
	MgO						1.7	
	Ni_2_O_3_						2	
	ZnO						3.3	
	Fe_2_O_3_						9.2	
[112]	SiO_2_	Micrm.	134.4	54.52	37.5	22.1	10	Amine-functionalized silica nanoparticles are more effective than typical nanoparticles
			134.4	23.71	37.5	13	28	
[113]	SiO_2_	Carb.	140.2	68.5	NA.	NA.	7.7	Disjoining pressure of SiO_2_ was the main mechanism to remove the oil from the surface
[114]	SiO_2_	Sst	135.5	66	26.5	1.95	25.43	SiO_2_ is more effective for light oil reservoir
			130	101	28.3	7.3	14.55	
[115]	SiO_2_	Sst	NA.	NA.	NA.	NA.	5–35	Arrangement of silicon nanoparticle improves IFT
[116]	LHPN	Sst	87	28	28	7	21	Wettability is altered when polysilicon is adsorbed on the sandstone pore wall
[117]	TiO_2_/SiO_2_ NCs	Carb.	138	48	39	13.2	26	Trapped oil is mobilized by the nanocomposite
[118]	LHP	Sst	NA.	NA.	14.7	9.3	2	Nanofluid was more effective for secondary recovery
[119]	TiO_2_	Sst	NA.	NA.	23	18	14	Low concentration of TiO_2_ improved the oil recovery
[117]	Fe_2_O_3_/SiO_2_ NC	Sst	138	52	39	17.5	31	Nanocomposite was able to alter wettability of the rock surface dramatically
[120]	SiO_2_	Sst	74	1.2	16	1.4	33	SiO_2_ can desorb the oil from the rock
[121]	TiO_2_	Sst	125	90	NA.	NA.	31	Higher disjoining pressure as a result of using higher concentration
[122]	SiO_2_	Micrm.	100	0	NA.	NA.	8.7 (0.1 wt%)	Contact angle and IFT are dependent on the weight % of nanoparticle
							26 (0.3 wt%)	
[123]	TiO_2_	Sst	18	8	47.5	44.5	9.5–13.3	Decrease in capillary force
[124]	ZrO_2_ and NiO	Carb.	152	44	NA.	NA.	NA.	Additional IFT reduction after using nanoparticle
[125]	Al_2_O_3_	Sst	131	92	38.5	2.25	20.2	Dispersant agent (propanol) was used for the first time and was effective in IFT reduction
	Fe_2_O_3_		132.5	101	38.5	2.75	17.3	
	SiO_2_		134	82	38.5	1.45	22.5	
[126]	SiO_2_	Carb.	NA.	NA.	NA.	NA.	8.7	By using nanoparticles, rheological properties of the displacing phase improved
**Nano-Surfactant**								
[127]	SDS + Al_2_O_3_	Carb.	92	75	9.88	2.75	NA	Anionic surfactant is less effective than cationic surfactant for carbonate reservoir
	SDS + ZrO_2_		92	84	9.88	2.78		
[128]	NaCl + CAPB + SiO_2_	Carb.	156.2°	75.1°	39.63	1.10	12.2	Decrease of IFT from 39.63 to 1.10 mN/m leads to oil improvement
[129]	SDS + SiO_2_	Micrm.	73	11	NA	NA	13	Extra heavy oil recovery as compared to SDS alone
[130]	3.22 ZrO_2_ + 0.50 g of CTAB	Carb.	180	32	NA	NA	10	Positive outcome is observed by surfactant and nanoparticle synergism
[131]	rhamnolipid BS-spherical + silica	Carb.	112	8	NA	1.85	26.1	Spherical shape nanoparticle is more effective than other shape nanoparticle due to uniformity
	rhamnolipid BS-sponge + silica		120	17	NA	1.94	25.1	
[132]	ZrO_2_ + SDS	Carb.	152	44	NA	NA	8	From the tests, it was obvious that ZrO_2_ is very effective in changing the wettability from oil wet limestone to water wet
[133]	SiO_2_ + ALFOTERRA	Carb.	167	146	23.2	7.2	10	Using nano was effective in additional oil recovery in ambient and HPHT conditions
[134]	Anionic surfactant + Al_2_O_3_	Carb.	142	0	NA.	NA.	NA	At relatively low concentrations, Al_2_O_3_ can improve anionic surfactant to alter the oil wet to water wet more effectively
[129]	A_2_O_3_/SiO_2_ + SDS, CTAB	Carb.	73	11	NA	NA	15	Small size and high surface area of nanoparticles were very effective
[127]	CTAB + Al_2_O_3_	Carb.	70	52	8.46	1.65	NA	Smaller particle size of Al_2_O_3_ leads to higher surface energy, resulting in bigger repulsion force
	CTAB + ZrO_2_		70	60	8.46	1.85		
[135]	SDS + ZrO_2_	Carb.	NA	NA	48	10	NA	At and below CMS nanoparticles have a great role in IFT reduction
[136]	non-ferrous metal + anionic surfactant	Sst	23	19	31.4	9.2	12–17	Nanoparticles decrease surfactant adsorption
[27]	ZrO_2_ + SDS	Carb.	101	30	16	3.1	25	Cationic surfactant was more effective at altering the wettability
	ZrO_2_ + CTAB		101	16	18.4	5.4	32.5	
[64]	Cationic anionic + silica	NA	59	46	45	43	45	Nanoparticle size 5–75 was effective at reducing the IFT
[137]	SDS + ZnO	Sst	NA	NA	32.5	7.1	19	Sodium dodecyl sulphate gives better stability of ZnO
[35]	ZnO + SDBS	Sst	44.45	42.47	10.86	10.2	8.5–10.2	Decreasing in NP size leads to contact angle reduction
[138]	SiO_2_ + Soloterra964	Sst	43.4	103.2	13.78	0.78	17.23	Nanosurfactant was a suitable EOR agent
[127]	TX-100 + Al_2_O_3_	Carb.	85	62	9.13	2.55	NA.	For carbonate, nonionic surfactant is more effective in altering the wettability as compared to ionic surfactant
	TX-100 + ZrO_2_		85	71	9.13	2.64	NA.	
**Nano-Polymer**								
[139]	SiO_2_ + 2-Poly(MPC)	Sst	NA.	NA.	47	35	5.2	Using copolymer with nano silica yielded higher oil recovery
[140]	Silica + DMAEMA	Sst	85	62.2	27	14	9.9	Nanoparticles reduce polymer adsorption
[141]	Nanoclay + HPAM	Sst	NA.	NA.	NA.	NA.	5	Improvement in viscosity after using Nanoclay/HPAM
[142]	SiO_2_ + PAM	Sst	NA.	NA.	27	10.2	24.7	Due to disjoining pressure, oil wet is changed to water wet
[143]	SiO_2_ + Xanthan gum	Sst	86	20	17.8	6.4	7.81	More oil is produced from unswept areas leading to improving residual oil recovery
[144]	SiO_2_ + PVP	Sst	54	22	19.2	7.9	0–6.1	Oil recovery increases with increasing the concentration of nanoparticles due to improved adsorption ratio
[98]	Al_2_O_3_ + PVP	Sst	54	21	NA.	NA.	7–24	IFT and contact angle improved synergistically, in a nanocomposite form as compared to individual nanoparticle
**Nano-SP**								
[62]	SiO_2_+ PAM + SDS	Sst	NA.	NA.	NA.	0.13	17.49	Pressure drop increased to 0.38 MPa
[62]	Clay + PAM + SDS	Sst	NA.	NA.	NA.	0.238	18.28	Higher viscosity as compared to conventional SP
[64]	Nanoclay + 2800 PAM + 0.2 SDS	Micrm.	NA.	NA.	NA.	NA.	6.8	The injection of nano to SP leads to a more uniform flow pattern in a micromodel, which yields a more stable front
	Nanoclay + 3000 PAM + 0.2 SDS						8	

LHPN = lipophobic and hydrophilic polysilicon nanoparticle; HLPN = hydrophobic and lipophilic polysilicon nanoparticle; NWPN = neutral wet polysilicon nanoparticle; TX-100 = Triton X-100; PVP = Povidone: Polyvinylpyrrolidone; DMAEMA = Dimethylamino-ethyl methacrylate; CTAB = Cetyl trimethylammonium bromide; CAPB = Cocamidopropyl betaine. MPC = methacryloyloxyethyl phosphorylcholine.
